# Repair of a perforated duodenal diverticulum using intraduodenal suture in 94 year old woman: A case report

**DOI:** 10.1016/j.ijscr.2020.04.083

**Published:** 2020-05-16

**Authors:** Hidenori Maki, Yasuhiro Yuasa, Yuta Matsuo, Osamu Mori, Atsushi Tomibayashi

**Affiliations:** Department of Surgery, Tokushima Red Cross Hospital, Tokushima, Japan

**Keywords:** Diverticulum, Duodenal suture, Perforation, Case report

## Abstract

**Introduction:**

In case of gastrointestinal diverticula, the duodenal type is quite frequent; duodenum is the second most common site for diverticula following the colon (Glener et al., 2016). However, duodenal diverticular perforation is rare, so the appropriate surgical treatment for this condition is yet unclear (Simoes et al., 2014). This literature has been written in line with the SCARE criteria (Agha et al., 2018).

**Presentation of case:**

A 94-year-old woman emergently presented to our department with diffuse abdominal tenderness and guarding, indicating pan-peritonitis. Computed tomography revealed pneumoretroperitoneum; Therefore, gastrointestinal perforation was suspected.

Emergency surgery was performed; we detected the perforated diverticulum located at the posterior wall of the duodenum with accompanying inflammation. Considering the location, postoperative leakage, and the patient's advanced age, we placed trans-section on duodenal wall against the lesion and resected the diverticulum, The lumen was then manually sutured from within. The anterior wall was closed using linear staplers. The patient recovered uneventfully and was discharged 24 days after surgery.

**Discusssions:**

As perforated duodenal diverticulum is rare, the ideal management is unclear. Several studies mention various surgical procedures. However, actual treatment varies based on the patient's situation and surgeon's assessment. Almost all cases that require surgery present emergently; therefore, simpler and more effective treatment methods are required (Simoes et al., 2014; Philip et al., 2019; Fujisaki et al., 2014).

**Conclusion:**

We used a unique surgical technique; intraduodenal suture for perforated diverticulum. This technique can be a feasible alternative for managing this condition.

## Highlights

• Duodenal diverticula are common; however, perforation is rare.
• No standard protocol exists for the management of perforated duodenal diverticula.
• Especially in emergent cases, a rapid and simple technique seems more feasible.
• We treated a woman with perforation using partial manual sutures inside the duodenum.
• This promises to be a feasible technique for managing such cases.

## 1 Introduction

While duodenal diverticulum are common, they have a low morbidity rate of approximately 5%; diverticula are primary diagnosed using radiographic imaging. Almost all cases are asymptomatic, unless complications like perforation develop. The estimated incidence of said complications is 0.03% per year [5]. Due to this, reports on surgical treatment for this condition are comparatively rare.

Some studies report various surgical procedures depending on the case, but definite management protocol is missing, especially for challenging cases. We report a case of duodenal diverticulum perforation that was successfully repaired using a novel surgical method. This report has been written in line with the SCARE criteria [6].

## 2 Presentation of case

**Fig. 1:**
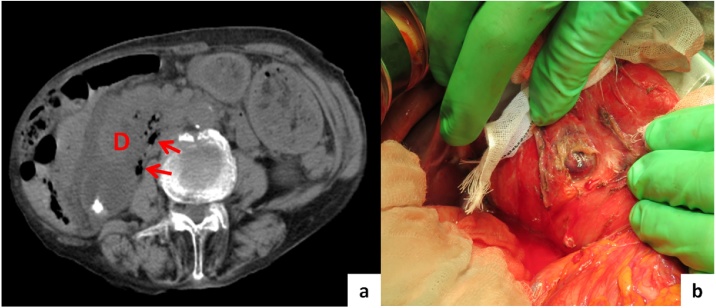
a: Retroperitoneal free air (arrows) around the duodenum (D). b: Perforated diverticulum at posterior wall of the duodenum.

The patient was a 94-year-old woman, who was almost fully independent in her daily life and had no signs of dementia. She presented at another hospital with sudden epigastric tenderness. Non-enhanced abdomino-pelvic computed tomography (CT) revealed free air located retroperitneally around the duodenum, indicating pan-peritonitis (Fig. 1a). Therefore, the patient was transported to our hospital for emergency operation.

Her vital signs were temperature, 37.9 °C; heart rate, 101 beats per minute; and blood pressure, 150/77 mmHg. All laboratory data and urinalysis were within the normal ranges and she had no appreciable change and medication.

Considering the findings of CT (Fig. 1a) and her symptoms, we decided to perform emergency surgery.

### 2.1 Surgical procedure

**Fig. 2:**
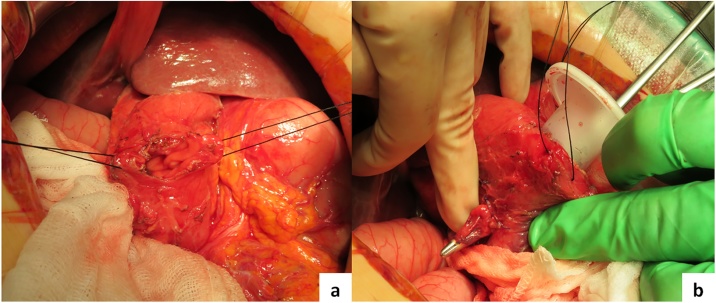
a: Section at the anterior duodenal wall against the leision. b: Using Lap-protector® and checked perforated site.

We made a 12 cm median incision to explore the upper gastric cavity. Minimal superficial ascites was seen at the perforated site. The second and third portions of the duodenum were red and inflamed, suggesting perforateion of the posterior wall of the duodenum. To better observe this lesion, Kocher’s maneuver was performed; after mobilization of the duodenum, we found the perforated deverticulum with a small stone (1 cm) inside at the posterior wall of the second portion of the duodenum. Inflammation and phlegmon extending to the retroperitoneum (Fig. 1b) were also seen. The perforation partially extended to the pancreas head; therefore it was difficult to close the perforated site due to its location, concerns about postoperative leakage, and the patient’s advanced age. The pancreas was slightly damaged, so we placed intraduodenal sutures in 3–4 cm section at the duodenal anterior wall against the lesion in the direction of the long axis of the duodenum. We explored inside the duodenum from the anterior window using a Lap protector mini® retractor on the edge of the window. We then found the perforated diverticulum and the involved stone (Fig. 2).

The bile flow was from the proximal side of the lesion, which was consistent with our estimate that Vater’s papilla was not involved.

**Fig. 3:**
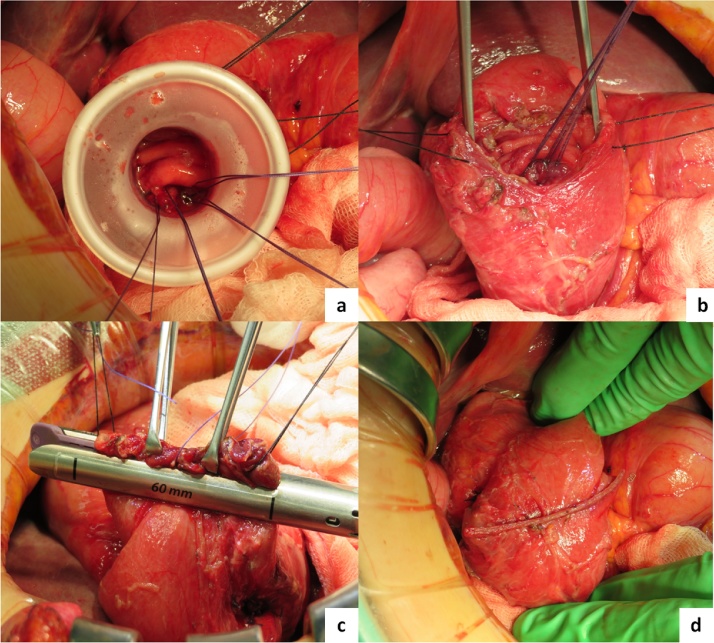
a: Pulled up the sutured diverticulum into the intraduodenal cavity. b: Manually sutured perforated site in the cavity. c,d: Closure of the window of anterior wall of the duodenum with GIA®.

We used 3-0 Vicryl® (Polyglactin 910 Suture VIOLET BRADED) to suture the diverticulum, pulled it up into the intraduodenal cavity and resected the diverticulum. We then manually sutured the perforated hole with the same piece of suture material followed by closure of the window on the anterior wall of the duodenum with an automatic suture device (GIA purple Tri-Staple™ technology 60mm®) in the direction of the short axis of the duodenum (Fig. 3).

### 2.2 Postoperative course

**Fig. 4:**
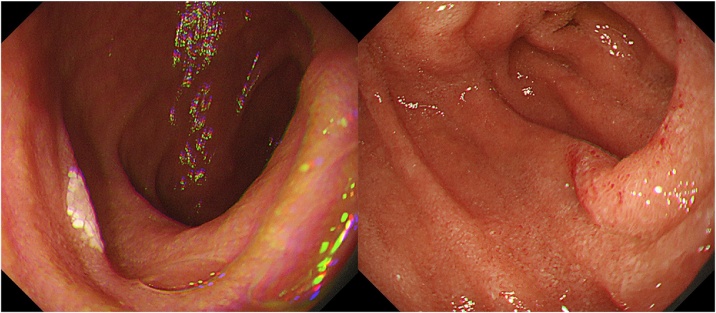
Intraduodenal cavity 2 months after surgery.

**Fig. 5:**
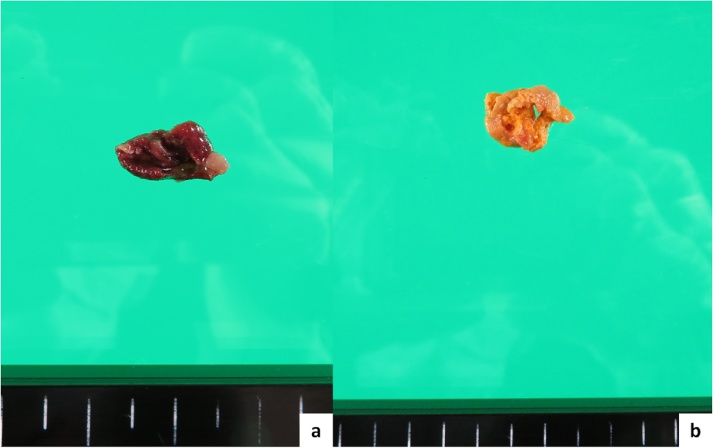
a: Resected diverticulum, no malignancy. b: Stone involved the diverticulum.

The patient’s postoperative course was uneventful. She was administered antibiotix (cefmetazole sodium 1 g/8 h) for 4 days and intravenous proton pump inhibitor for 7 days after surgery. She fully recovered and was discharged 24 days after surgery. Gastrointestinal endoscopy (Fig. 4) and CT 2 months after surgery revealed a completely cured perforation site. The final pathological diagnosis was perforated duodenal diverticulum with no malignancy; however, we could not determine whether the resected diverticulum was ture or larvate (Fig. 5).

## 3 Discussion

Duodenal diverticulum is generally asymptomatic; while complications are rare (0.03% per year [5]). They include hemorrhage, fistula, compression of other organs, inflammation, and perforation. Although perforation is the rarest complication, it has mortality rate of 20–30% [1,2]. Treatment for perforated duodenal diverticulum is unclear as the condition is rare. While report on surgical (open or laparoscopic), conservative (antibiotics or drainage),and conversion treatments are available, on reviewing these reports, we found that treatment strategies for severe cases were based on the patient’s symptoms, vital signs, age, tolerance for surgery, and laboratory data; in other words, there is no standard treatment [5].

In terms of surgical intervention, simple diverticulectomy followed by closure is the most frequent procedure; if retroperitneal inflammation is present, more complex procedures like segmental resection, duodenal diversion, pyloric exclusion, gastro-enteric anastomosis, or pancreaticoduodenectomy(PD) might be necessary [3,4,7]. In two-thirds of cases the duodenal diverticulum perforates into retroperitneum; therefore in emergent operations, surgeons face difficulties even if it is a simple diverticulotomy [7]. In this case, we faced a challenging situation because the pancreas was inflamed. Although PD was an alternative, we realized that it is highly invasive and the patient’s physical tolerance was low due to her advanced age. While we determined PD to be too risky, drainage only was deemed insufficient. We therefore performed “intra-luminal diverticulectomy” and sutured the duodenal wall using interrupted sutures.

The procedure we performed was less risky and required less time compared to PD and was more effective than only drainage. The Lap protector® was very useful because it enabled excellent visualization of surgical field. We were also concerned about possible damage to Vater’s papilla; therefore, we checked bile flow from the proximal side after opening anterior wall of the duodenum and did not find Vater’s papilla, which revealed our concern. We also observed the patient for post- operative leakage or pancreatitis, but she recovered without any complications. To the best of our knowledge, this technique; has not been used before. The technique has its limitations. The procedure needs to be completed rapidly. The comparison risks and benefits needs to be judged for each individual patient based on the characteristics of the case. However, it is universally accepted that complicated procedures should be avoided in emergency situations; our procedure provides a simpler and faster alternative for perforated duodenal diverticulum.

## 4 Conclusion

Perforated duodenal diverticulum with inflammation extending to the retroperitoneum or pancreas poses a challenge regarding closure of the perforated site, especially in emergent situations. Intra-duodenal sutures are a simpler and more feasible alternative. However, more cases needs to be studied for data accumulation.

## Declaration of Competing Interest

We don’t have no conflicts of interest to declare on this literature.

## Sources of funding

There is no funding sources.

## Ethical approval

The ethical approval was given from our institution.

## Consent

Our patient and family has signed a consent form.

## Author contribution

Hidenori Maki : Operating Surgeon and writing the paper.

Yasuhiro Yuasa : Assistant Surgeon, Supervisor and writing the paper.

Mizuki Fukuta : Writing the paper.

Taihei Takeuchi : Writing the paper.

Takao Tsuneki: Writing the paper.

Keisuke Fujimoto: Writing the paper.

Yuta Matsuo : Writing the paper.

Osamu Mori : Writing the paper.

Shohei Eto : Writing the paper.

Satoshi Fujiwara : Writing the paper.

Atsushi Tomibayashi : Writing the paper.

Kazumasa Okumura : Writing the paper.

Hisashi Ishikura : Writing the paper.

## Guarantor

Hidenori Maki.

## Provenance and peer review

Not commissioned, externally peer-reviewed.
